# Triage of Women with Minor Cervical Lesions: Data Suggesting a “Test and Treat” Approach for HPV E6/E7 mRNA Testing

**DOI:** 10.1371/journal.pone.0012724

**Published:** 2010-09-13

**Authors:** Sveinung Wergeland Sørbye, Silje Fismen, Tore Gutteberg, Elin Synnøve Mortensen

**Affiliations:** 1 Department of Clinical Pathology, The University Hospital of North Norway, Tromsø, Norway; 2 Department of Microbiology and Infection Control, The University Hospital of North Norway, Tromsø, Norway; 3 Department of Medical Biology, Faculty of Health Sciences, University of Tromsø, Tromsø, Norway; Karolinska Institutet, Sweden

## Abstract

**Background:**

Human papillomavirus (HPV) testing is included in the cervical cancer screening program in the triage of women with equivocal (ASC-US) or low-grade (LSIL) cytological lesions. These women have an increased risk for developing high grade dysplasia and cancer (CIN2+) compared to women with normal cytology. However, in order to avoid unnecessary follow-up, as well as overtreatment, a high positive predictive value (PPV) of the triage test is important.

**Methodology/Principal Findings:**

The HPV test PreTect HPV-Proofer, detecting E6/E7 mRNA from the HPV types 16, 18, 31, 33 and 45, is used as triage test together with repeat cytology. PPV data for HPV E6/E7 mRNA testing during the period from January 2006 up to June 2009 are reported. In total, 406 of 2099 women (19.3%) had a positive HPV test result. Of the women with a positive test result and with a histological diagnosis (n = 347), 243 women had histological high-grade dysplasia or cancer (CIN2+), giving a PPV of 70.0% (95% confidence interval [CI], 65.2%–74.8%). For HPV 16 or HPV 33 positive women above 40 years of age, the PPV was 83.7% (95% CI, 73.3%–94.0%) and 84.6% (95% CI, 65.0%–100.0%) respectively. The PPV of test positive women with HSIL cytology was 94.2% (95% CI, 88.7%–99.7%).

**Conclusions:**

When the result in triage is HPV mRNA positive, our data suggest direct treatment for women above 40 years of age or for women with a concurrent cytological HSIL diagnosis, contributing to better clinical safety for these women. In addition, by decreasing the time to treatment, thereby reducing the number of recalls, the patient management algorithm will be considerably improved, in turn reducing follow-up costs as well as unnecessary psychological stress among patients.

## Introduction

Cervical cancer can be prevented by early detection and treatment of precancerous lesions. In Norway, a cervical cancer screening program was introduced in 1995, recommending all women between 25 and 69 years of age to have a cytological cell sample (Pap-smear) every three years. Since the introduction of the program, the coverage of women taking a Pap-smear has increased and consequently, the rate of cervical cancer is reduced [1]. Despite this well organized screening program, about 300 women get cervical cancer in Norway each year (age adjusted cancer incidence of 8.9 per 100 000 person-years) [2] about half of whom are following the program [1]. This can be explained by a generally low clinical sensitivity of cytology and by subjectivity and poor reproducibility [3]. These limitations are mainly a concern in primary screening, but are also a concern in secondary screening where cytology is used to triage women with equivocal or low-grade cellular changes. However, in secondary screening, women with cellular changes are already found and will be closely followed up with the methods available. It is therefore important to identify women with true precancerous lesions or with cervical cancer, i.e. women requiring immediate treatment. In addition, it is important to avoid overtreatment by detecting as few false positives as possible [4], [5]. Considering all of the above, this has drawn attention to the need for objective and accurate diagnostic tests providing additional information in order to identify women having a true risk of developing cancer.

**Figure 1:**
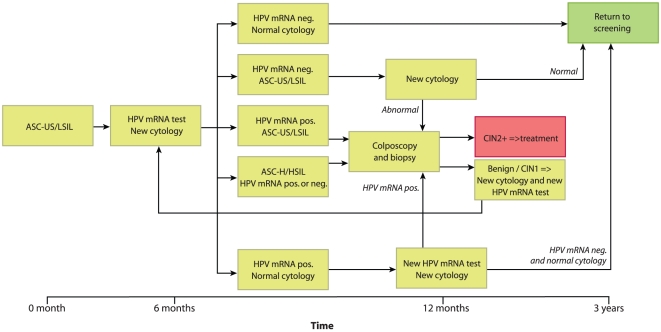
Flow chart showing the guidelines for HPV E6/E7 mRNA testing. Women with ASC-US or LSIL at triage and with a positive HPV result are referred to colposcopy and biopsy; if the HPV test is negative, the woman is referred to a third Pap-smear after one year. Women with normal cytology and a positive HPV mRNA result at triage are recommended to have a third Pap-smear and a new HPV mRNA test after 6 months; women with a persistent HPV mRNA result are referred to colposcopy and biopsy. Women with ASC-H and HSIL at triage are referred directly to histological investigation regardless of HPV status.

As a result of the finding that human papillomavirus (HPV) is a necessary cause of cervical cancer, HPV testing has been included in cervical cancer screening in several countries. In Norway, HPV testing has been included in secondary screening since 2005 (Figure 1): If the cytology is defined as ASC-US (atypical squamous cells of undetermined significance) or LSIL (low-grade squamous intraepithelial lesions), the woman is referred to a second Pap-smear (repeat cytology) and an HPV test after six months. If the repeat cytology reveals ASC-US or LSIL and the HPV test is positive, the woman is referred to colposcopy. Women with normal repeat cytology but with a positive HPV result are tested for HPV persistence and if positive, referred to colposcopy. Women with ASC-H or HSIL are referred to colposcopy and biopsy independent of the HPV result.

In Norway, several HPV tests for use in secondary screening are available. One of these tests is the PreTect HPV-Proofer assay (NorChip AS, Norway), detecting HPV E6/E7 mRNA from the five most prevalent types causing cervical cancer. The University Hospital of North Norway has five years of experience with the use of this test. The argument for choosing this test in secondary screening was the need for a clinically specific test to find the women truly needing follow-up and essentially requiring referral to treatment. The HPV E6/E7 mRNA test has been shown to have a higher clinical specificity and positive predictive value (PPV) than HPV DNA tests [6]–[12] and thus, in our opinion, qualifies better than HPV DNA tests for this purpose.

The PPV reflects the proportion of patients with a positive test result who are correctly diagnosed and gives information about the accuracy of the test. At our hospital we have repeatedly observed that women with a cytological normal, ASC-US or LSIL lesion at triage, and who are HPV mRNA positive, actually have an underlying high-grade lesion or cancer which is revealed at colposcopy and biopsy. In addition, re-evaluation of the cytological or histological slides after a positive HPV test often reveals more severe cell-changes than first diagnosed. In a previous paper [13] we report our general results using the HPV mRNA test in secondary screening (reporting frequency of the different HPV types, clinical sensitivity, specificity, PPV and negative predictive value (NPV)). Here, we focus on the PPV of the test, including more comprehensive data.

## Materials and Methods

### Ethics Statement

The Regional Committee for Research Ethics has approved the study. Written consent from the patients for their information to be stored in the hospital database and used for research was not needed because the data were obtained and analyzed anonymously.

In the routine diagnostic practice at the University Hospital of North Norway, the HPV E6/E7 mRNA test PreTect HPV-Proofer (detecting E6/E7 mRNA from the HPV types 16, 18, 31, 33 and 45) is used in the triage of women with an ASC-US or LSIL diagnosis for the detection of cervical dysplasia and cancer. The Department of Clinical Pathology receives cervical smears from the population of Troms and Finnmark County in Northern Norway. Approximately 23 000 cervical smears are analyzed each year and from January 2006 to June 2009, smears from 54 326 women aged 25–69 years were analyzed. A total of 2 858 women (5.3%) were diagnosed with ASC-US or LSIL. For these women, repeat cytology and the HPV mRNA test were recommended after six months. Liquid based cytology and HPV mRNA results were received from 2 099 women (73.4% of the 2 858 women). The reason for not obtaining HPV mRNA results for all the cases is the wide use of conventional Pap-smear rather than liquid based cytology, which does not allow for HPV analysis.

Cells were extracted from the ThinPrep® 2000 (Cytyc Corporation, Marlborough, MA, USA) for cytological examination. The HPV mRNA test uses a liquid based sample from the same material as cytology. The mRNA testing was performed according to the manufacturer's instructions (NorChip AS, Klokkarstua, Norway) and in accordance with national guidelines for HPV testing (Figure 1).

Cytological and histological diagnoses were obtained from the diagnostic database (SymPathy) at the Department of Clinical Pathology, University Hospital of North Norway. Biopsies with histological high-grade dysplasia or cancer (CIN2+) were evaluated by experienced pathologists. In Norway, the current clinical threshold for treatment is CIN2+; after histologically confirmed CIN2+, national guidelines recommend treatment by conization of the cervix. Women with benign or CIN1 histology are advised to be followed up with a new Pap-smear and HPV test after 6–12 months. Biopsies with uncertain cellular changes are immunostained with p16 in order to detect occult CIN lesions.

### Limitations of the study

It is important to note that in clinical practice, the guidelines are not consistently followed. In contrast to a well planned study, individual evaluations are frequently made and essentially the history of the patient is important in the assessment of whether or not she should be referred to treatment. Different aspects of deviations are included in the discussion. Furthermore, due to ethical considerations, conization of every woman is not possible and therefore the true sensitivity of the test cannot be determined.

## Results

Numbers for PPV calculations are presented in Tables 1–3. Of the 2 099 women who were tested for HPV mRNA, 406 (19.3%) had a positive test result. Not unexpectedly HPV 16 was the most dominant type, detected in 205 (50.5%) of the positive women, followed by HPV 33 (16.0%), HPV 18 (13.8%), HPV 45 (13.5%) and HPV 31 (6.2%) (Table 2). Of the 406 women with a positive HPV result, histological data were available for 347 women.

**Table 1:** Triage of women with ASC-US or LSIL.

Cytological diagnosis at triage	Number of women with this diagnosis	HPV positives	Women with CIN2+ among the HPV positives	Number of women with a histological diagnosis	PPV* (95% CI)
**Normal**	1 191	42	13	20	65.0 (44.1–85.9)
**Unsatisfactory**	109	16	6	9	66.7 (35.9–97.5)
**ASC-US**	358	98	45	89	50.6 (40.2–60.9)
**AGUS**	2	0	-	-	-
**LSIL**	252	115	64	97	66.0 (56.6–75.4)
**ASC-H**	103	63	50	63	79.4 (69.4–89.4)
**HSIL**	84	72	65	69	94.2 (88.7–99.7)
**Total**	2 099	406	243	347	70.0 (65.2–74.8)

*PPV for CIN2+ for HPV mRNA test positives. Only women with a histological diagnosis (biopsy or cone) are included in the calculation.

**Table 2 pone-0012724-t002:** PPV for CIN2+ for the different HPV-types, including all ages.

HPV type	Number of women with respective HPV type	Women with CIN2+ among the HPV positives	Number of women with a histological diagnosis	PPV* (95% CI)
**16**	205	144	179	80.4 (74.6–86.3)
**18**	56	25	50	50.0 (36.1–63.9)
**31**	25	13	21	61.9 (41.1–82.7)
**33**	65	37	56	66.1 (53.7–78.5)
**45**	55	24	41	58.5 (43.5–73.6)
**Total**	406	243	347	70.0 (65.2–74.8)

*Only women with a histological diagnosis (biopsy or cone) are included in the calculation.

**Table 3:** PPV for CIN2+ for the different HPV-types, including women >40 years of age.

HPV type	Number of women with respective HPV type	Women with CIN2+ among the HPV positives	Number of women with a histological diagnosis	PPV* (95% CI)
**16**	55	41	49	83.7 (73.3–94.0)
**18**	22	10	19	52.6 (30.2–75.1)
**31**	8	3	6	50.0 (10.0–90.0)
**33**	17	11	13	84.6 (65.0–100.0)
**45**	18	6	14	42.9 (16.9–68.8)
**Total**	120	71	101	70.3 (61.4–79.2)

*Only women with a histological diagnosis (biopsy or cone) are included in the calculation.

For women being treated (n = 230), the diagnosis used for PPV calculations is the most high-grade histological diagnosis of the biopsy before treatment or of the cone after treatment; the biopsy before treatment may have therapeutic effect and therefore the diagnosis of the biopsy may be the correct one. The diagnosis of the biopsy is reported for 42 women; for 181 women, the diagnosis of the cone is reported. Seven women were referred to treatment directly after abnormal cytology and/or colposcopy and/or a persistent HPV result and for these women the diagnosis of the cone is reported.

Of the total women with a positive HPV result and a histological diagnosis (n = 347), 243 women had histological CIN2+, giving a PPV of 70.0% (Table 1). Of the HPV 16 positive women, 144 of 179 women with histology had a diagnosis of CIN2+, giving a PPV of 80.4% (Table 2). Due to the lower rate of planned children among women >40 years of age, the data for this age group were analyzed separately (Table 3). For women planning for children, cervical surgery should, even with a high PPV of the confirmatory test, always be given higher consideration.

The added value of the HPV mRNA test in detecting women with high-grade lesions is shown in Table 4 and 5. Table 4 summarizes the number of women with a normal, ASC-US or LSIL diagnosis at triage found to have an underlying high-grade lesion after a positive HPV test. Table 5 compares the diagnosis of the first biopsy with the most high-grade histological diagnosis for women referred to treatment. HPV mRNA positive women with benign or low-grade changes at first biopsy are always advised to be followed with a new Pap-smear and HPV mRNA test after six months. However this follow-up is commonly inconsistently performed (for example, only a conventional Pap-smear is taken, not allowing for HPV testing). Thus, only women referred to treatment are included in the table.

**Table 4:** Added value of the HPV test; number of HPV mRNA test positives having an underlying high-grade lesion not detected at triage cytology.

HPV type	CIN2				CIN3				CxCa			
	N	UN	ASC-US	LSIL	N	UN	ASC-US	LSIL	N	UN	ASC-US	LSIL
**16**	5	0	7	19	3	2	16	20	0	0	2	2
**18**	2	0	2	3	0	0	1	4	0	0	1	0
**31**	0	1	1	1	0	1	1	0	0	0	0	0
**33**	1	0	6	4	1	1	4	6	0	0	0	0
**45**	1	1	4	3	0	0	0	2	0	0	0	0

N  =  Normal.

UN  =  Unsatisfactory.

**Table 5:** HPV mRNA positive women with definite treatment: Diagnosis of first biopsy compared to the most high-grade histological diagnosis.

Diagnosis of first biopsy	Most high-grade histological diagnosis					
	Benign	CIN1	CIN2	CIN3	CxCa	Total
**No biopsy** 1)	1	0	2	4	0	7
**Benign** 2)	0	1	2	6	0	9
**Uncertain** 2)	0	1	0	1	1	3
**CIN1** 2)	0	7	4	9	0	20
**CIN2**	0	0	70	33	3	106
**CIN3**	0	0	0	76	8	84
**CxCa**	0	0	0	0	1	1
**Total**	1	9	78	129	13	230

1) Women referred to treatment directly after a positive HPV result and/or high-grade cytology and/or a previous CIN2+ diagnosis (with or without treatment).

2) HPV positive women with histology <CIN2 at first biopsy were advised to be followed up with a new Pap-smear and mRNA test after 6 months. Women were referred to new biopsy and/or treatment on the basis of a persistent HPV infection and/or a HSIL diagnosis at follow-up. Alternatively, the same biopsy may have been re-evaluated on the basis of a positive HPV result and then revealing a high-grade lesion.

In total, 13 women were found to have cancer (Table 5), all of whom were either HPV 16 or 18 positive. Five of these women had an ASC-US or LSIL diagnosis at cytology (Table 4). All but one had either microinvasive carcinoma or stage 1A carcinoma; one patient had stage 1B carcinoma. Nine women have undergone hysterectomy and for four women (aged 26, 29, 32 and 33 years), conization (possibly with re-conization) was sufficient for eliminating further disease.

## Discussion

The main cause of invasive cervical cancer is the deregulated and persistent production of HPV E6 and E7 oncoproteins [14]. Hence, HPV E6/E7 mRNA is a rational target for detecting HPV infections leading to cellular transformation. PreTect HPV-Proofer is a commercial assay for the detection of E6/E7 mRNA from the five main high-risk HPV types 16, 18, 31, 33, and 45. Due to the higher clinical specificity and PPV of this method compared to other HPV tests [6]–[12], this was the method of choice at our hospital when HPV testing was introduced in secondary screening in Norway.

Our data show that when including all age groups, the HPV mRNA test has a PPV for CIN2+ of 70%. A previous data analysis [13] shows that the PPV of repeat cytology for CIN2+ is 29.8% (unpublished data; this number is overrated due to known HPV status). In a meta-analysis by Marc Arbyn and colleagues [15], a PPV of repeat cytology of 11.8% for ASC-US and 23.2% for LSIL is reported; the PPV of repeat cytology at cutoff ASC-US or worse ranged from 3.8% to 22.2%. Taken together, our results indicate that the PPV of the HPV mRNA test strongly exceeds that of repeat cytology, which is an important finding. The 30% with a positive HPV test but with no confirmatory high-grade lesion may be explained by the observation that colposcopic biopsy misses 26–42% of prevalent CIN2+ (depending on the severity of the lesion and the number of biopsies taken) (Marc Stoler, Eurogin 2010), with the main problem being the subjectivity in determining the location of the lesion [16]. The question then arises whether the 30% of the HPV mRNA positive cases with no confirmatory high-grade histological diagnosis actually may represent false negatives due to a small lesion or a biopsy not representing the true lesion. Also, HPV 18 infections have been described as being specifically difficult to detect histologically and may therefore lead to a false negative histological diagnosis [17]. Interestingly, in several cases we have demonstrated that women with an originally benign or low-grade histological diagnosis have underlying lesions when the biopsy is more thoroughly examined after a positive HPV mRNA test result (for example by making deeper sections of the biopsy or by p16 immunostaining) indicating that, in several cases, a false negative histological result was revealed.

The PPV of the diagnoses ASC-H and HSIL for CIN2+ are, according to Norwegian Cancer Registry data, 56.4% and 73.9% respectively [1]. The respective numbers from our lab are 52.7% and 76.9% (unpublished data; first cytology 2005–2008, CIN2+ up to end 2008). The data in the present study show that by including HPV E6/E7 mRNA testing, the PPV of test positive women with ASC-H or HSIL is 79.4% and 94.2%, respectively. This strongly suggests that women with HSIL and a positive HPV mRNA test can be directly referred to treatment. For women with ASC-H, the HPV test will be a valuable tool in separating women requiring follow-up (or treatment if >40 years of age or not planning for children) from women only having a chronic inflammation (seen in more than 40% of women with ASC-H). Women with HSIL and a negative HPV mRNA test should be referred to colposcopy and histology in the traditional way.

Women with ASC-US or LSIL at repeat cytology and with a positive HPV mRNA test are referred directly to colposcopy and biopsy. When an HPV test is not used, women with ASC-US and LSIL are referred to colposcopy and biopsy after three times repetition of the diagnosis. This means that for women with an underlying high-grade lesion, the disclosure of their disease will be delayed with up to two years, which may be critical. The same applies to women with normal cytology at triage, for whom the PPV of HPV mRNA test positives for CIN2+ is 65.0%. Women with normal cytology (when the HPV test is not included) are referred back to the screening program, emphasizing the importance of an HPV test for these women. Of note is that the PPV for mRNA positive women with normal cytology is higher than the PPV for women with ASC-US (Table 1). This may be explained by small sample size and overlapping confidence intervals. It may also be explained by the fact that for women with normal cytology, the guidelines recommend biopsy only when a persistent infection is demonstrated (HPV positive with six months interval) and consequently the risk of having a CIN2+ is higher.

An HPV mRNA positive HPV 16 result has a PPV for CIN2+ of 80.4% when including all age groups, representing a higher PPV than the cytological HSIL diagnosis. For the other HPV types, the PPV is somewhat lower. One explanation for the lower PPV of HPV 18 and HPV 45 may be the lower degree of noticeable cell changes related to these infections and the fact that lesions caused by these HPV-types often are located further up in the cervical canal (glandular lesions) [17]. Therefore, for these infections, if the original biopsy is benign or low-grade, diagnostic conization might be more important than for infections with other HPV types.

The motivation for looking at the age group >40 years is related to the lower probability of children planned among women in this group. Given the impact of cervical treatment (such as LEEP conization) on pregnancy outcomes [5], [18], [19], cervical treatment is more critical for women planning to have children. In addition, if a high-grade lesion is found in a woman above 40 years of age, there is a higher chance that the lesion has been present for a longer period of time, in turn making surgery more important. Accordingly, for women above 40 years of age, the PPV of 70.3% (83.7% for HPV16 and 84.6% for HPV 33) suggests that HPV mRNA test positive women can be referred directly to treatment without histological confirmation, contributing to a better clinical safety for this age group. For the 30 women above 40 years of age with no confirmed high-grade lesion, 14 had a negative HPV test at follow-up (out of a total of 15 women receiving a follow-up HPV test; one had a persistent HPV 45 infection). Fifteen women were followed up by cytology only, and of these, three had ASC-US, one had LSIL and 11 had at least one normal cytological smear and were consequently referred back to the screening program. However, these women may have lesions further up in the cervical canal that are difficult to detect morphologically, as often seen for HPV 18 or 45 infections. In our case, nine of these 15 women had either an 18 or 45 infection. We would therefore suggest that all women with a positive HPV result should be followed up with a repeated HPV test after six months. Also, among postmenopausal women, a higher degree of atrophic changes are present, often being difficult to distinguish from dysplasia. For these women, an HPV test will be crucial for the evaluation of the sample and subsequent follow-up of the woman.

Tables 1 and 2 show that for 59 out of 406 women with a positive HPV mRNA test, no histological diagnosis is reported. There may be several reasons for this. First of all, for women with a normal cytological diagnosis at triage, referral to biopsy is recommended only if they are found to have a persistent HPV-infection or a HSIL at follow-up. A new HPV test is only performed for about half of these women, due to misunderstandings related to the new strategies for clinical management or to the fact that a conventional Pap-smear is taken, not allowing for HPV testing. Some times a new cytological smear is done, although a biopsy has been requested (for example after a persistent HPV result). If this smear is found to be normal, the woman may disappear from the system and is a typical example of a woman “lost in follow-up”. Another reason for lack of histological data may be that the woman is pregnant and a biopsy is not recommended for this group. Alternatively, the woman may have moved to another city and consequently the follow-up of this woman has been done elsewhere.

An added value of the HPV mRNA test in detecting women with high-grade lesions is presented in Table 4, among others showing that five women with an ASC-US or LSIL diagnosis at triage had underlying carcinoma revealed after a positive HPV test. Another added value of the HPV test is seen for women with a benign or low-grade diagnosis at first biopsy. If the HPV test is positive, a new Pap-smear and HPV test are recommended after six months. In our material, 23 women with a benign, uncertain or CIN1 diagnosis at first histology had an underlying high-grade lesion revealed during follow-up after a positive HPV result (Table 5). For these women, the first biopsy or histological slide may not have been representative for the underlying disease. Alternatively, a microlesion difficult to detect by colposcopy-directed biopsy or by histology may have been present. Nevertheless, this in turn emphasizes the need for additional methods in order to increase the overall sensitivity of biopsy and histology.

In total, 13 women (with ASC-US, LSIL or HSIL at triage) were diagnosed with cancer. Interestingly, the HPV test had an important role in detecting most of these women, explained by the knowledge that the cytological diagnosis is influenced by the HPV result and by the follow-up of women with normal or low-grade cytology due to a positive HPV result (Table 4). Consequently, the HPV mRNA testing led to an earlier diagnosis and better prognosis: All but one of these women had a final diagnosis of either microinvasive carcinoma or stage 1A carcinoma; one patient had stage 1B carcinoma. Nine women had hysterectomy and for four women, conization was sufficient to eliminate further disease.

To conclude, our data suggest a clinical “test and treat” application of the HPV mRNA test: Direct treatment may contribute to a better clinical safety for test positive women above 40 years of age and for women with a concurrent cytological HSIL diagnosis. Consequently, by decreasing the time between testing and treatment, thereby reducing the number of recalls, the patient management algorithm will be considerably improved and will in turn reduce follow-up costs and more importantly, unnecessary psychological stress among patients will be avoided.
